# Supplementary material for: The interactome of intact mitochondria by cross-linking mass spectrometry provides evidence for coexisting respiratory supercomplexes
Source: Mol Cell Proteomics. 2017 Dec 8;17(2):216–32. doi: 10.1074/mcp.RA117.000470 (PMC5795388; doi:10.1074/mcp.RA117.000470)
Supplement: Supplemental Data [file supp_RA117.000470_133922_0_supp_23978_fzffwf.zip › spectra_annotation/mito_spectra_annotation/72-1-1-1-1-1.pdf]

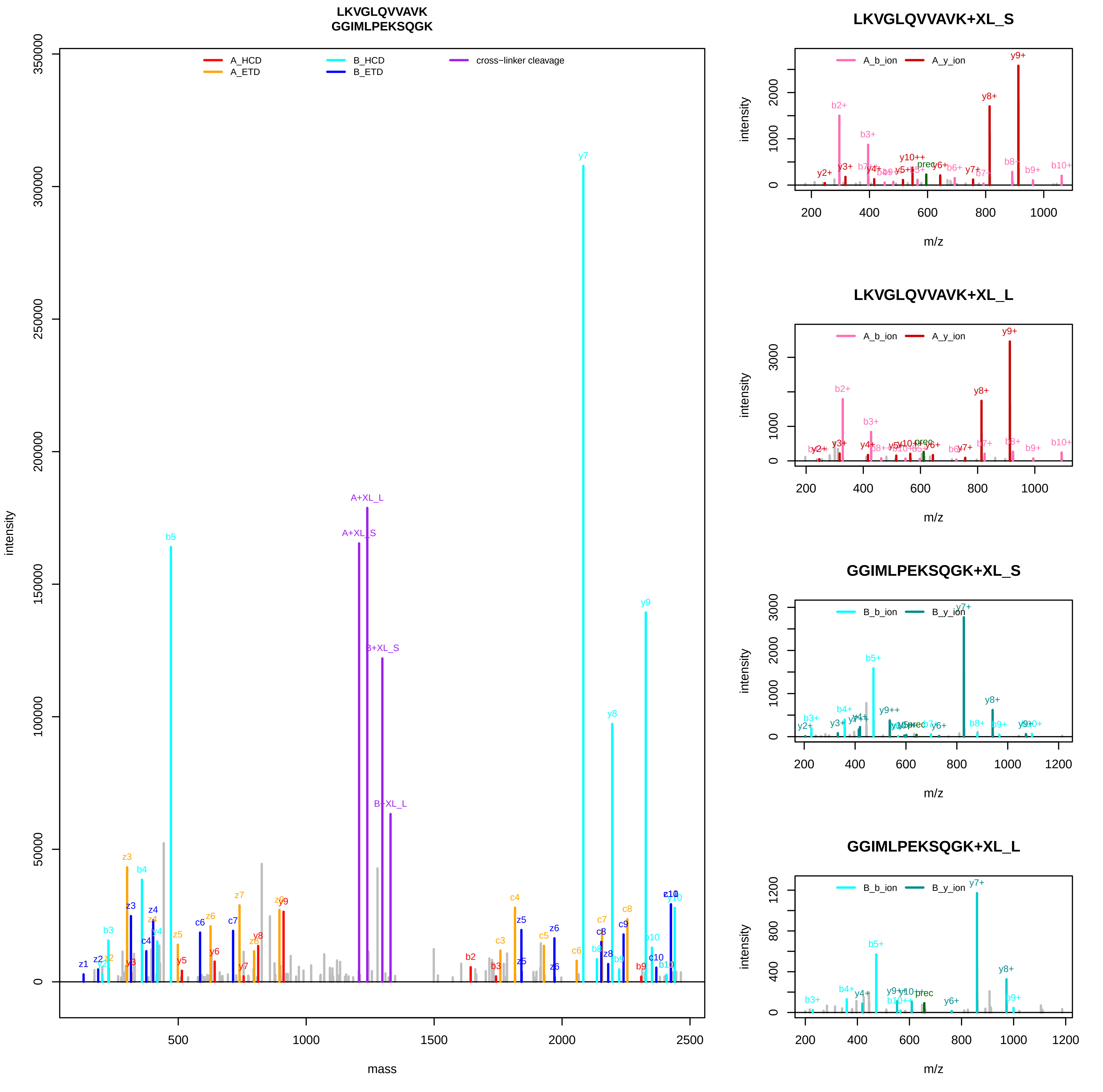
LKVGLQVVAVK
GGIMLPEKSQGK
A_HCD
A_ETD
B_HCD
B_ETD
cross−linker cleavage
intensity
mass
0
50000
100000
150000
200000
250000
300000
350000
500
1000
1500
2000
2500
y7
A+XL_L
A+XL_S
B+XL_S
B+XL_L
b5
y9
y8
z3
b4
b3
c4
c8
c10
y10
LKVGLQVVAVK+XL_S
LKVGLQVVAVK+XL_L
GGIMLPEKSQGK+XL_S
GGIMLPEKSQGK+XL_L
A_b_ion
A_y_ion
B_b_ion
B_y_ion
y9+
y8+
b2+
b3+
y7+
b5+
m/z
intensity
